# The exposure to uteroplacental insufficiency is associated with activation of unfolded protein response in postnatal life

**DOI:** 10.1371/journal.pone.0198490

**Published:** 2018-06-13

**Authors:** Annalisa Deodati, Josepmaría Argemí, Daniela Germani, Antonella Puglianiello, Anna Alisi, Cristiano De Stefanis, Roberto Ferrero, Valerio Nobili, Tomás Aragón, Stefano Cianfarani

**Affiliations:** 1 Dipartimento Pediatrico Universitario Ospedaliero, ‘Bambino Gesù’ Children’s Hospital–University of Rome Tor Vergata, Rome, Italy; 2 Division of Hepatology and Gene Therapy, Center for Applied Medical Research (CIMA), University of Navarra, Pamplona, Spain; 3 Department of Systems Medicine, University of Rome Tor Vergata, Rome, Italy; 4 Hepatometabolic Unit, ‘Bambino Gesù’ Children’s Hospital, Rome, Italy; 5 Department of Women’s and Children’s Health, Karolinska Institutet, Stockholm, Sweden; Wayne State University, UNITED STATES

## Abstract

Early life events are associated with the susceptibility to chronic diseases in adult life. Perturbations of endoplasmic reticulum (ER) homeostasis activate the unfolded protein response (UPR), which contributes to the development of metabolic alterations. Our aim was to evaluate liver UPR in an animal model of intrauterine growth restriction (IUGR). A significantly increased expression of X-box binding protein-1 spliced (XBP1s) mRNA (p<0.01), Endoplasmic Reticulum-localized DnaJ homologue (Erdj4) mRNA (p<0.05) and Bip/GRP78-glucose-regulated protein 78 (Bip) mRNA (p<0.05) was observed in the liver of IUGR rats at birth. Furthermore, the expression of gluconeogenesis genes and lipogenesis genes were significantly upregulated (p<0.05) in IUGR pups. At 105 d, IUGR male rats showed significantly reduced glucose tolerance (p<0.01). A significant decreased expression of XBP1s mRNA (p<0.01) and increased expression of double-stranded RNA-dependent protein kinase-like ER kinase (PERK) and Asparagine synthetase (ASNS) (p<0.05) was observed in the liver of IUGR male adult rats. Liver focal steatosis and periportal fibrosis were observed in IUGR rats. These findings show for the first time that fetal exposure to uteroplacental insufficiency is associated with the activation of hepatic UPR and suggest that UPR signaling may play a role in the metabolic risk.

## Introduction

Early life events play a critical role in the long-term susceptibility to chronic diseases [1]. Epidemiological studies have shown an association between low-birth weight and cardiometabolic risk, including visceral adiposity, hypertension, dyslipidemia, insulin resistance, glucose intolerance, type 2 diabetes and cardiovascular disease [2,3].

To explain this association the *thrifty phenotype* hypothesis was proposed [4].

According to this hypothesis, when the fetus is exposed to malnutrition the organism diverts the limited nutrient supply to favor the survival of vital organs, such as brain, at the expense of growth and other organs, such as liver and pancreas. This fetal adaptation to in utero undernourishment leads to permanent endocrine and metabolic changes through postnatal life, eventually predisposing to cardiometabolic risk [5].

The process by which insults occurring at critical periods of development, lead to permanent changes in organ function is known as intrauterine programming [6], whose underlying molecular mechanisms have not been elucidated yet.

Functional deficiencies in the endoplasmic reticulum (ER), the intracellular organelle responsible for maturation and folding of most membrane and secreted proteins [7], have been identified as a hallmark of metabolic and inflammatory disorders. In conditions characterized by overwhelming of ER protein folding capacity, misfolded proteins accumulate in the lumen of the ER membranous network, and trigger the activation of a set of intracellular signalling molecules that mediate the unfolded protein response (UPR). A wide variety of stimuli, including glucose and/or nutrient deprivation, viral infections, lipid overload, increased synthesis of secretory proteins, and expression of mutant or misfolded proteins have been reported to activate UPR [8–10].

In mammals, UPR signaling is initiated from three independent transmembrane ER stress sensors: inositol-requiring 1 α (IRE1 α), double-stranded RNA-dependent protein kinase-like ER kinase (PERK) and activating transcription factor 6 (ATF6α and β). Under physiological, non-stress conditions, these transducers are kept in an basal state by the binding with the chaperone glucose-regulated protein 78 (Bip/GRP78) [11,12]. The endoribonuclease activity of IRE1α cleaves a 26 base-pair non canonical intron within the mRNA encoding the X-box binding protein-1 (XBP1) transcription factor. Ligation of the resulting exons yields an spliced transcripts that, in turn, is translated to produced spliced XBP1 protein (XBP1s). The concerted actions of XBP1s/ATF6/ATF4 establish a transcriptional program aimed to restore ER homeostasis.

In liver, folding and secretion of large quantities of protein (millions of molecules per minute) [13] require an expanded ER network and a precise control of ER homeostasis. Beyond protein folding control, hepatic UPR signaling is closely linked to the regulation of lipid and carbohydrate metabolism.

The growing interest for the involvement of ER stress in the pathophysiology of metabolic disorders originates from the observation that UPR signalling mechanisms are exacerbated in the liver and adipose tissue of obese rodents [7]. In humans, this finding has been confirmed in liver and subcutaneous adipose tissue of obese patients [14,15].

The aim of this study was to investigate the UPR activation in an animal model of intrauterine growth restriction (IUGR) induced by uteroplacental insufficiency, from birth to adulthood.

## Materials and methods

### Animal model

The animal model of intrauterine growth restriction (IUGR) induced by uteroplacental insufficiency was previously described [16]. In brief, time-dated Sprague-Dawley pregnant rats (Harlan-Envigo, Udine, Italy) were individually housed under standard conditions and were allowed free access to standard chow diet and water. On day 19 of gestation (term is 22 d), maternal rats were anesthetized with intramuscular injections of xylazine (8 mg/kg) and ketamine (40 mg/kg) (Sigma-Aldrich, St.Louis, MO), and the abdomen was opened along the midline. Suture was placed around both the uterine arteries and then either tied or withdrawn before closing the abdomen. In our study we have included only the dams that delivered within 24h on GD22. We excluded the few dams who delivered before or after GD 22. In our study we have included only the dams that delivered within 24h on GD22. We excluded the few dams who delivered before or after GD 22. Dams recovered quickly from uterine artery ligation and SHAM procedures, and resumed feeding the same day. The animals started moving 4 hours after surgery and were kept warm by paper towels during recovery. After recovery, rats had *ad libitum* access to food and water. The pregnant rats were allowed to deliver spontaneously, and approximately 8 h after delivery, pups were weighed and the litter size was randomly reduced to eight to assure uniformity of litter size between IUGR and SHAM litters. At birth, 14 SHAM and 14 IUGR pups were decapitated. The remaining pups were fostered to unoperated normal female rats and remained with their foster mothers until they were weaned. At 105 post-natal days (PND) rats were sacrificed by cervical dislocation. Animal cages were kept isolated to avoid animal stress. Analgesia was not used to avoid drug absorption in the harvested tissues sacrificed were immediately harvested, frozen in liquid nitrogen, and stored at -80°C. 10 SHAM/11 IUGR males rats and 13 SHAM/8 IUGR females rats from four different litters per each study group were randomly selected.

The study protocol was approved by the Committee for Animal Research of Tor Vergata University, Rome, Italy (Ministry of Health, Department of Veterinary Public Health approval 6/12/2012, #153/2001-A). Animal experiments were performed according to the Guide for the Care and Use of Laboratory Animals of the National Institutes of Health (NIH Publication No. 85–23, revised 1996). All procedures complied with Italian regulations for laboratory animal care, according to the guidelines and under supervision of the Animal Technology Station, Interdepartmental Service Center, Tor Vergata University, Rome, Italy.

### Plasma assays

Blood samples were obtained quickly after sacrifice by cardiac puncture, collected in tubes and centrifuged at 3000 r/min for 10 min. at 4°C. Serum was collected and stored at −80°C. Glucose was determined using a colorimetric commercial kit (Sigma-Aldrich).

Plasma insulin concentrations were measured in duplicate by a rat/mouse insulin enzyme-linked immunosorbent assay (ELISA) kit, using rat insulin as the standard (Millipore Co.Vimodrone, Italy) according to the manufacturer’s instructions. The intra-assay coefficient of variability (CV) was 1.17–3.22%, the inter-assay CV was 6.71–9.23%, and the sensitivity limit was 0.1 ng/mL.

Leptin concentrations were measured using a leptin ELISA kit (R&D Systems, UK) according to the manufacturer’s instructions. The intra-assay CV was 3.8–4.3%, the inter-assay CV was 5–7.6%, and the sensitivity limit was 22 pg/mL. Quantification of free fatty acids (FFA) was performed using a commercially available ELISA kit (Bioassay Tech. Lab., China) according to the manufacturer’s instructions. The intra-assay CV was <10%, inter-assay CV was <12%, sensitivity was 2.51 mM/L.

### Oral glucose tolerance test

The animals were fasted for 4 hours before sacrifice and plasma samples were collected between 11 am to 1 pm.

For Oral glucose tolerance test (OGTT), rats were fasted for 6 hours and gavage fed with glucose (2 g/kg body weight). Glucose levels were measured both before and 15, 30, 60 and 120 min. after glucose administration by OneTouch UltraEasy (Life Scan, California).

### RNA isolation and cDNA synthesis

Total RNA from 20 mg of liver tissue was extracted using the automated Maxwell system (Promega, Madrid, Spain) according to the manufacturer’s instructions and quantified in duplicate using UV absorbance at 260 nm. One microgram of RNA, pre-treated with RNase free DNase, was transcribed into the cDNA using the MLV-RT (Promega) in a final volume of 40 μL following the manufacturer’s protocol. To minimize variation in the reverse transcription reaction, all RNA samples from a single experimental setup were reverse transcribed simultaneously.

### Real-time quantitative polymerase chain reaction

PCR primers to amplify different genes are described in supplemental methods (S1 Table). Ribosomal protein large P0 (*Rplp0*) was used as an internal normalization control. Experiments were performed in duplicate using 96-well tray and optical adhesive covers (Bio-Rad) in a final reaction mixture of 10 μL containing 1 μL of undiluted cDNA. Real-time PCR was performed using SybrGreen (Bio-Rad) on iQ5 (Bio-rad, Madrid, Spain). The cycling consisted of 2 min. at 50°C, 2 min. at 95°C followed by 40 cycles of 95°C for 15 s and 60°C for 45 s. Determination of reaction efficiency was routinely used as an internal quality control for adequate assay performance. Results are expressed in raw relative quantification+/-standard errors.

### Total liver protein extracts and western blot analysis

Frozen liver fragments (50 mg) were homogenized in 500 μL of homogenization buffer: [7M Urea, 2M Thiourea, 4% CHAPs, 40 mM DTT, supplemented with protease and phosphatase inhibitors (Complete and Phostop, respectively; Roche, Mannheim, Germany)] and ultracentrifuged at 75.000 rpm for 45 min. Supernatants were collected in a clean cryo-tube and conserved in -80°C until use. Equivalent amounts of protein were separated in denaturing polyacrylamide gels and transferred onto a nitrocellulose membrane. The primary antibodies against p-eIF2α (Ser51-#9721), IRE1α (#3294), Irβ (#3025), AKT(#9272), p-AKT (Thr308-#9275) and p-AKT (Ser473-#9271) were purchased from Cell Signaling Technology, XBP1(sc7160) and EIF2α (sc11386) from Santa Cruz Biotechnology and ATF6 (IMG273) from IMGENEX, alpha-tubulin (T6074) from Sigma. Detection of immunolabeled proteins was performed using a commercial chemiluminescent assay (ECL prime; Amersham, Buckinghamshire, UK). Visualization and quantitative measurements were made with a CCD camera and software for Western blot image analysis (Odissey Fc Imager System and Image Studio Lite v 4.0, respectively; Li-COR, Bad Homburg, Germany).

### Liver histology

Hepatic tissues were fixed in 4% paraformaldehyde (PFA,Sigma-Aldrich) overnight at 4 degrees, followed by serial dehydration in 30, 50, 70 and 80% aqueous ethanol for 24 hours at each concentration, at room temperature (RT). Afterwards, samples were placed for 6 h in 96% ethanol, then in 99.6% ethanol and 100% butyl acetate, each overnight at RT and finally embedded in paraffin (Paraplast X-TRA, Sigma-Aldrich) at 61°C overnight. Paraffin-embedded tissue was cut to a thickness of 5 μm, using a Biocut sectioning machine (Reichert-Jung, NT, USA), mounted on microscope slides (Superfrost Plus, Thermo Scientific, MA, USA) and placed at 37°C overnight.

For histology, tissue sections were dewaxed with xylene (Histolab, Göteborg, Germany) for 10 min. and then serially rehydrated with 99.6, 96, and 70% aqueous ethanol, each step being performed twice for 5 min. Samples were subsequently stained with periodic acid-Schiff (PAS, Sigma-Aldrich). In brief, after washing twice with distilled water, samples were incubated for 5 minutes with periodic acid and then rinsed with tap water followed by 2x distilled water. Samples were then incubated for 15 min. with Schiff’s reagent and washed again as previously described. Slides were finally incubated with hematoxylin solution modified according to Gill III for 2 min., washed with tap water for 3 min., dehydrated with increasing aqueous ethanol solution and 100% xylene, and finally mounted with Entellan new (Merck, Vimodrone, Italy) and cover glass.

Images were captured using Zeiss Axio Imager M1 and photographed with a digital color camera system (Olympus DP70, Tokyo, Japan) attached to DP controller imaging software.

The PSR is intended for use in the histological visualization of collagen in tissue sections, Picro-Sirius Red (PSR) staining was performed with Connective Tissue Stain Kit (Abcam). The staining was performed on 2 μm-thick sections obtained from formalin-fixed tissue embedded in paraffin. The light microscopy imaging was performed on Nikon E600 light microscope equipped with NIS Elements BR software.

Histologic evaluation was performed based on the NAFLD Clinical Research Network (CRN) criteria [17].

### Statistical analysis

Differences in gene expression between SHAM and IUGR rats were analysed with one-way ANOVA. Differences between means from plasma assays and densitometric analyses were assessed by unpaired two-tailed t test. Differences were considered statistically significant at p < 0.05. All analyses were performed using SPSS version 13.0 for Windows (SPSS, Chicago, Illinois).

## Results

### Animal weights and metabolic profile at birth

**Fig 1:**
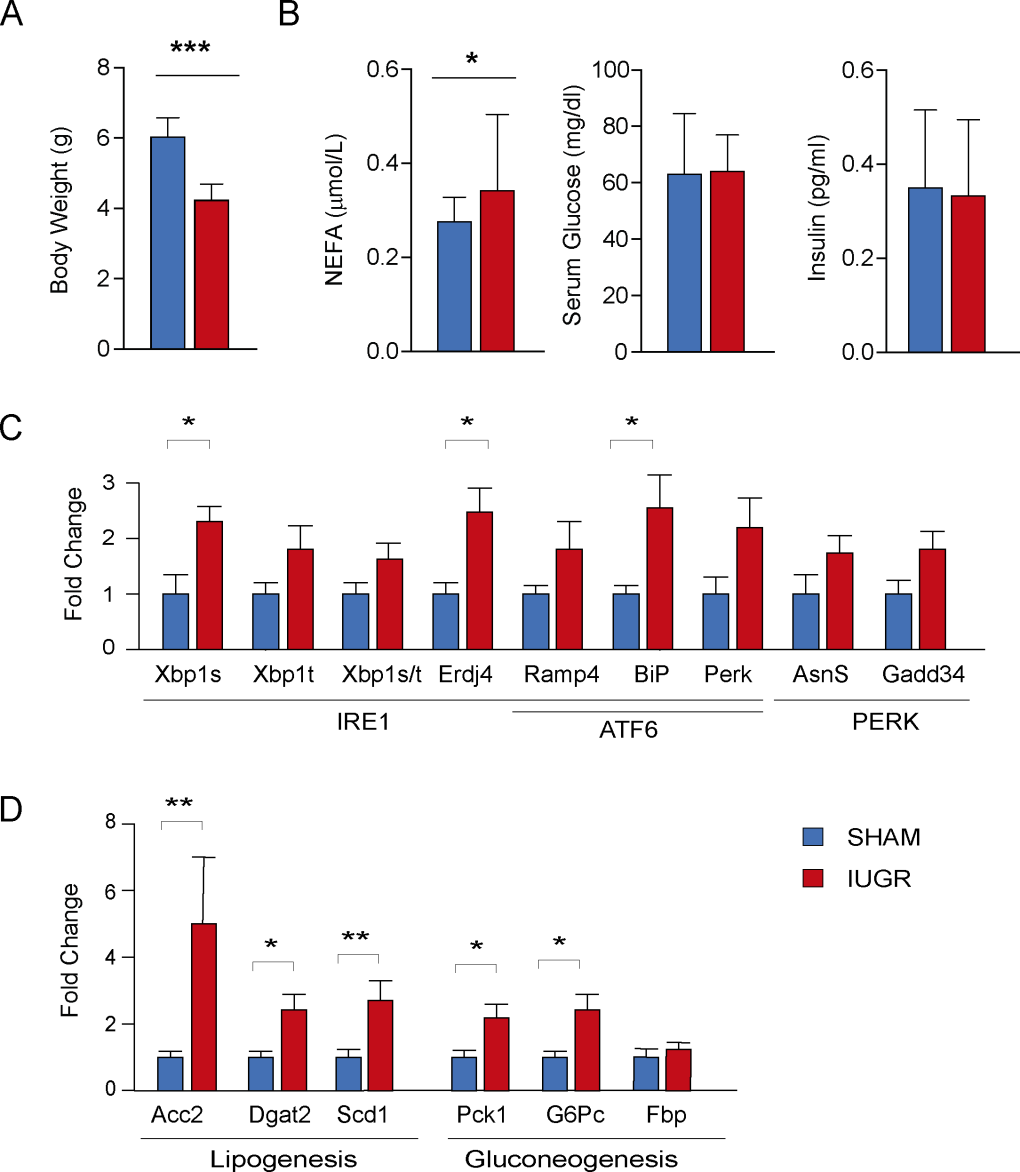
**A**. Birth weights of SHAM (n = 14) and IUGR (n = 14) animals (mean weight + SD, *p<0.001). **B**. Plasma concentrations of metabolic parameters in 14 SHAM and 14 IUGR (mean ± SD, *p<0.001). **C.** Expression of target genes from three branches of UPR (IRE1alpha, ATF6 and PERK). **D.** Expression of main lipogenesis and gluconeogenesis genes in liver. Values are expressed as fold change (mean + SD, *p<0.01, ** p<0.005).

This study was conducted in a well-established rat model of IUGR generated by the ligation of uterine arteries. One of the main hallmarks of intrauterine nutrient restriction is the reduced birth weight. As shown in Fig 1, the weight of IUGR rat pups at post- natal day 0 (PND0) was significantly reduced in comparison with SHAM animals (mean weight ± SD: 4.0±0.57 versus 6.5± 0.32 g; p<0.001, Fig 1A). Biochemistry revealed no significant differences in blood glucose (63.9±13 vs 62.9±21.5 mg/dl) and insulin (0.34±0.15 vs 0.36±0.16 ng/ml) levels among IUGR and SHAM animals. IUGR animals showed significantly higher serum free fatty acids (FFAs) levels (0.35±0.08 vs 0.28±0.05 mM/L; p<0.001) (Fig 1B).

### UPR activation in the liver of IUGR pups at birth

Next, we measured the levels of mRNAs produced in response to the different UPR mechanisms in the liver of male and female PND0 pups. Activation of the kinase/endonuclease IRE1α leads to the non-canonical splicing of the mRNA encoding the transcription factor XBP1. We observed that spliced XBP1 mRNA levels were significantly increased in IUGR rats (2.08 ± 1.22 vs 1 ± 0.88; p<0.01). Without reaching statistical significance, a similar trend was observed for total XBP1 mRNA, and for the spliced/total XBP1 mRNA ratio, suggesting that the increase in spliced XBP1 mRNA was due, at least in part, to the activation of IRE1α-mediated splicing. XBP1s mRNA encodes a potent transcription factor that enhances the expression of specific UPR target genes, such as the chaperone ERdJ4 mRNA, which was in fact significantly increased in IUGR rats (2.35 ± 1.43 vs 1 ± 0.7; p<0.05). The genes transcriptionally activated by the XBP1, ATF6 or ATF4 displayed a similar trend in IUGR liver samples, reaching statistical significance in the case of GRP78/BiP (2.3 ± 2.0 vs 1 ± 0.46; p<0.05) (Fig 1C).

Since IRE1α/XBP1 activation has been shown to promote transcription of genes involved in hepatic lipogenesis and gluconeogenesis, we monitored the steady-state mRNA levels of lipogenic and gluconeogenic enzymes. As shown in Fig 1D, a significant increase of mRNA levels of lipogenic enzymes Acetyl-CoA carboxylase 2 (Acc 2) (5.0 ± 7.6 vs 1.0 ± 0.3, p<0.01), diacylglycerol O-acyltransferase 2 (Dgat 2) (2.42 ± 2.0 vs 1.0 ± 0.31, p<0.05) and Stearoyl-CoA desaturase-1 (Scd 1) (2.70 ± 2.30 vs 1.0. ± 0.81, p<0.01) and the rate-limiting gluconeogenic enzymes Phosphoenolpyruvate Carboxykinase 1 (PCK1) (1.40 ± 0.6 vs 1.02 ± 0.15, p<0.05) and glucose-6-phosphatase catalytic subunit (G6Pc) (2.38±1.55 vs 1.0 ±0.46, p<0.01) was observed in IUGR pups. Of note, fructose 1, 6-bisphosphatase (FBP), one of the gluconeogenic limiting enzymes was not upregulated in IUGR animal (Fig 1D). These findings suggest an upregulation of the gene expression program for de novo glucose and lipid biosynthesis, associated with the raise in UPR sensor transcription, in liver of IUGR pups.

### Animal weights and metabolic profile in adult animals

**Fig 2:**
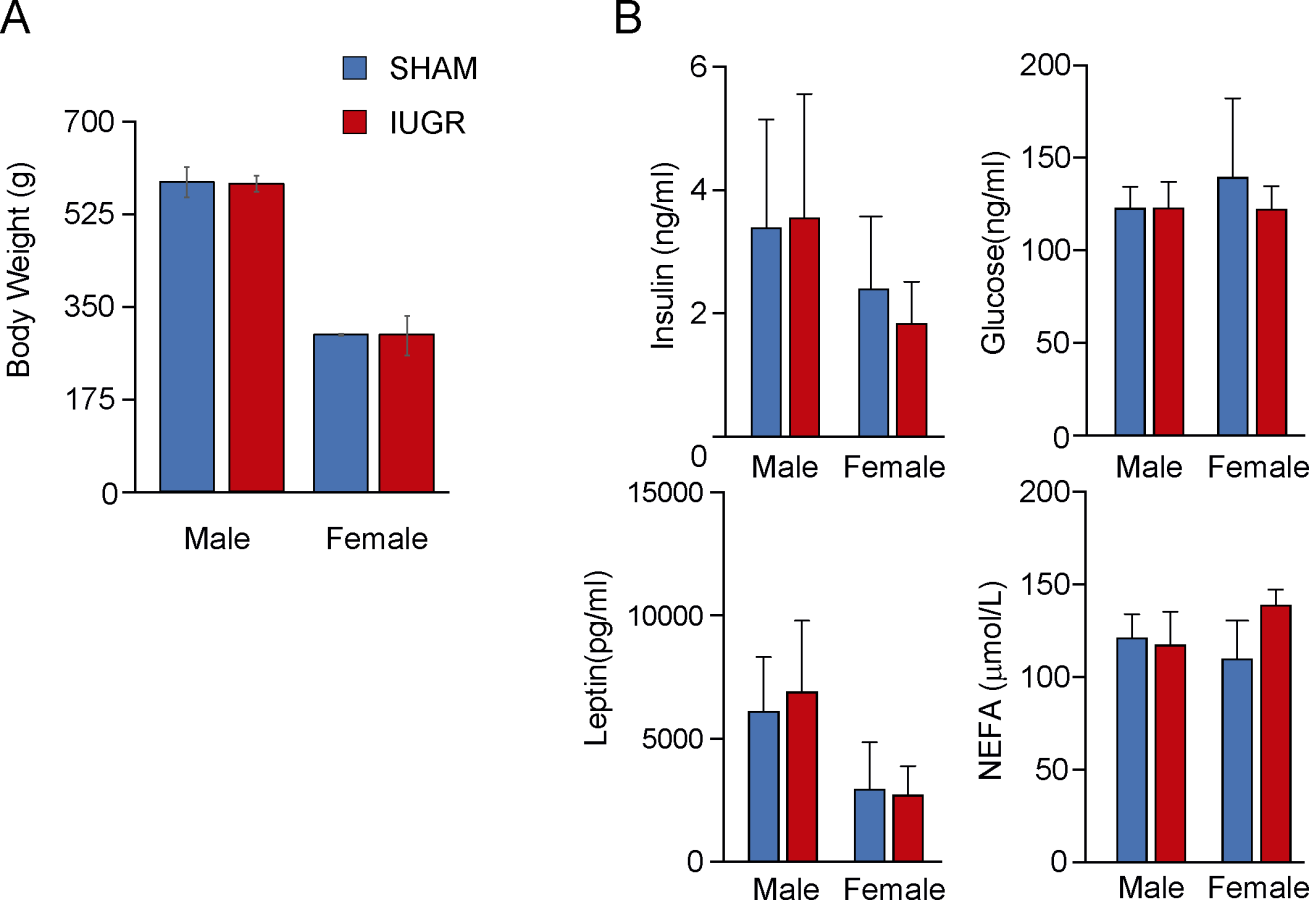
**A.** Weights of SHAM (n = 11) and IUGR (n = 11) at PND 105. **B** Plasma concentrations of metabolic parameters in 11 SHAM and 11 IUGR adult animals (mean + SD, **p<0.005).

At one hundred and five days after birth (PND105), IUGR rats showed no significant difference in weight when compared to SHAM animals (mean weight ± SD: 421.9±146.7 versus 443.76± 130.4 g, Fig 2A) and did not display significant differences in the blood levels of glucose, insulin, leptin or free fatty acids (FFAs) (Fig 2B).

**Fig 3:**
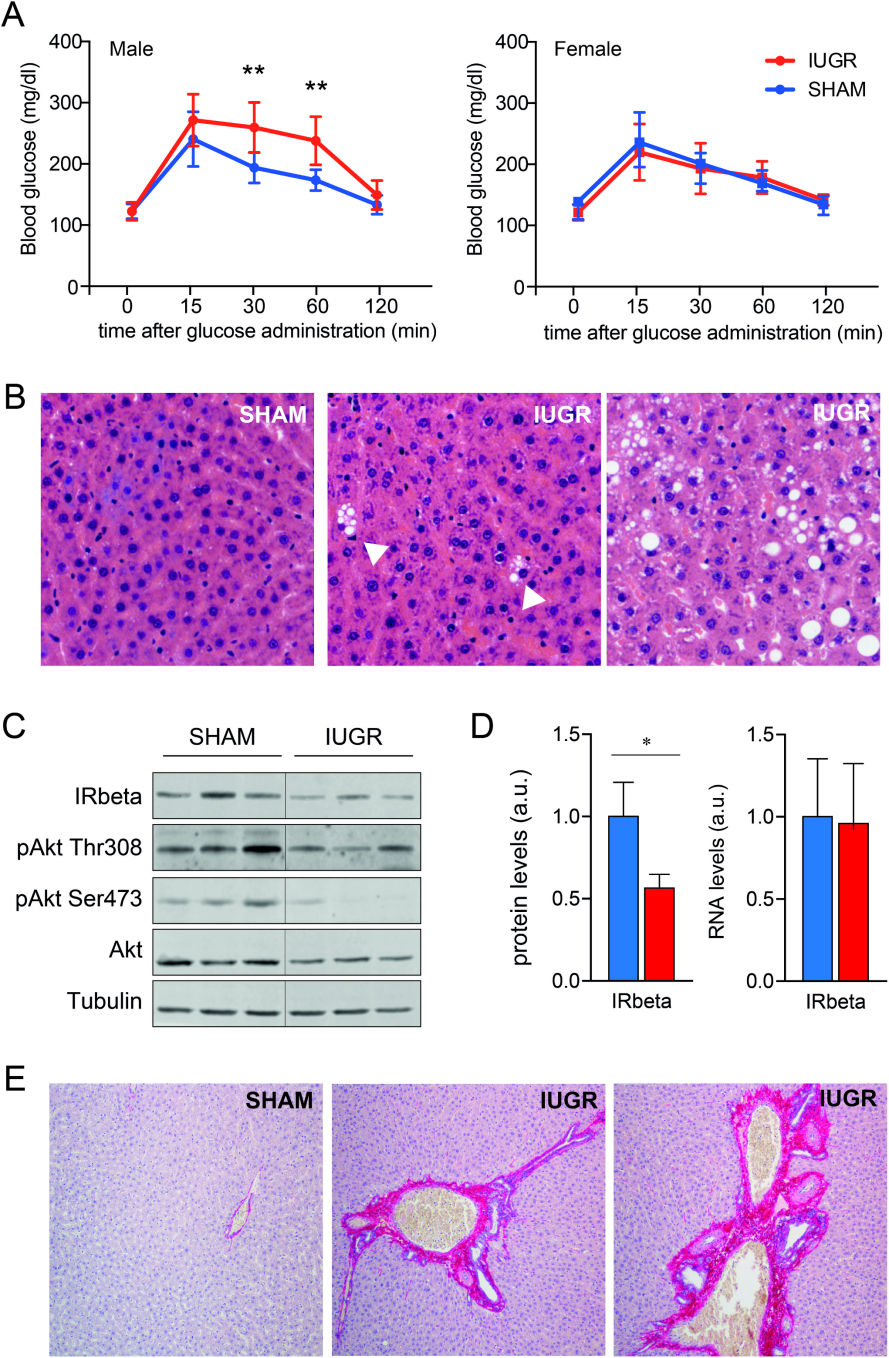
**A**. OGTT in male and in female rats at PND105 (mean ± SD, **p<0.005. **B.** Histology of SHAM and IUGR animals at PND105 (staining with H&E). **C.** WIB of insulin signaling in IUGR and SHAM adult animals. **D.** Protein and mRNA level of IR beta. **E.** Evaluation liver tissues of fibrosis pattern in IUGR and SHAM adult males rats, using Picro-Sirius Red Stain. Values are expressed as fold change (mean + SD, * p<0.01).

Oral glucose tolerance tests (OGTT) were carried out in PND105 rats. IUGR male rats showed significantly higher glucose levels during OGTT at 30 and 60 minutes (t 30’ 242.82 ± 38.7 vs 188.44 ± 19.44, p<0.01; t 60’ 217.7 ± 44.8 vs 171.7 ± 16.77, p< 0.05), whereas IUGR female rats did not show any significant difference in glucose tolerance (Fig 3A). Consistent with evidence in humans [18], the findings in the rat IUGR model recapitulate the influence of gender in the development of IUGR-associated insulin resistance.

### Insulin signaling in liver of adult animals

Due to the gender differences in glucose homeostasis observed at PND105, our next experiments focused on male rats. To assess whether the altered OGTT kinetics in IUGR animals was a bona fide surrogate of defective hepatic insulin signaling, we evaluated total and phosphorylated AKT, as well as insulin receptor beta in liver tissue. IUGR male rats showed lower levels of total and phospho- Ser473 AKT levels, as well as a clear reduction in insulin receptor beta subunit (IRbeta) (Fig 3C and 3D). This difference was most likely due to post-transcriptional regulation of these genes, as IRbeta mRNA levels were similar in IUGR and SHAM animals.

In liver, insulin signaling drives the expression of lipogenic enzymes, while it mitigates gluconeogenic transcription. To assess whether the reduced insulin signaling would lead to altered metabolic transcription, we analyzed the expression of gluconeogenic and lipogenic enzymes described above, without detecting any significant difference between IUGR and SHAM rats (S1 Fig).

### Liver histology

Since molecular and functional findings indicated that PND105 IUGR animals would be at an early stage of liver metabolic dysfunction, we performed liver histological analysis to evaluate hepatic steatosis or fibrosis. A scattered focal steatosis in IUGR male rats compared with SHAM animals was observed (Fig 3B). Furthermore, a pattern of periportal and perisinusoidal fibrosis (stage 2) was detected in the 100% of IUGR rats while only in 30% of SHAM animals (NAFLD Fibrosis Score = 2±0 vs 1.3±0.48, respectively, p<0.01, Fig 3E).

### UPR activation in the liver of IUGR males at PND105

RNA and protein analyses showed an unexpected differential activation of the different UPR signaling mechanisms.

**Fig 4:**
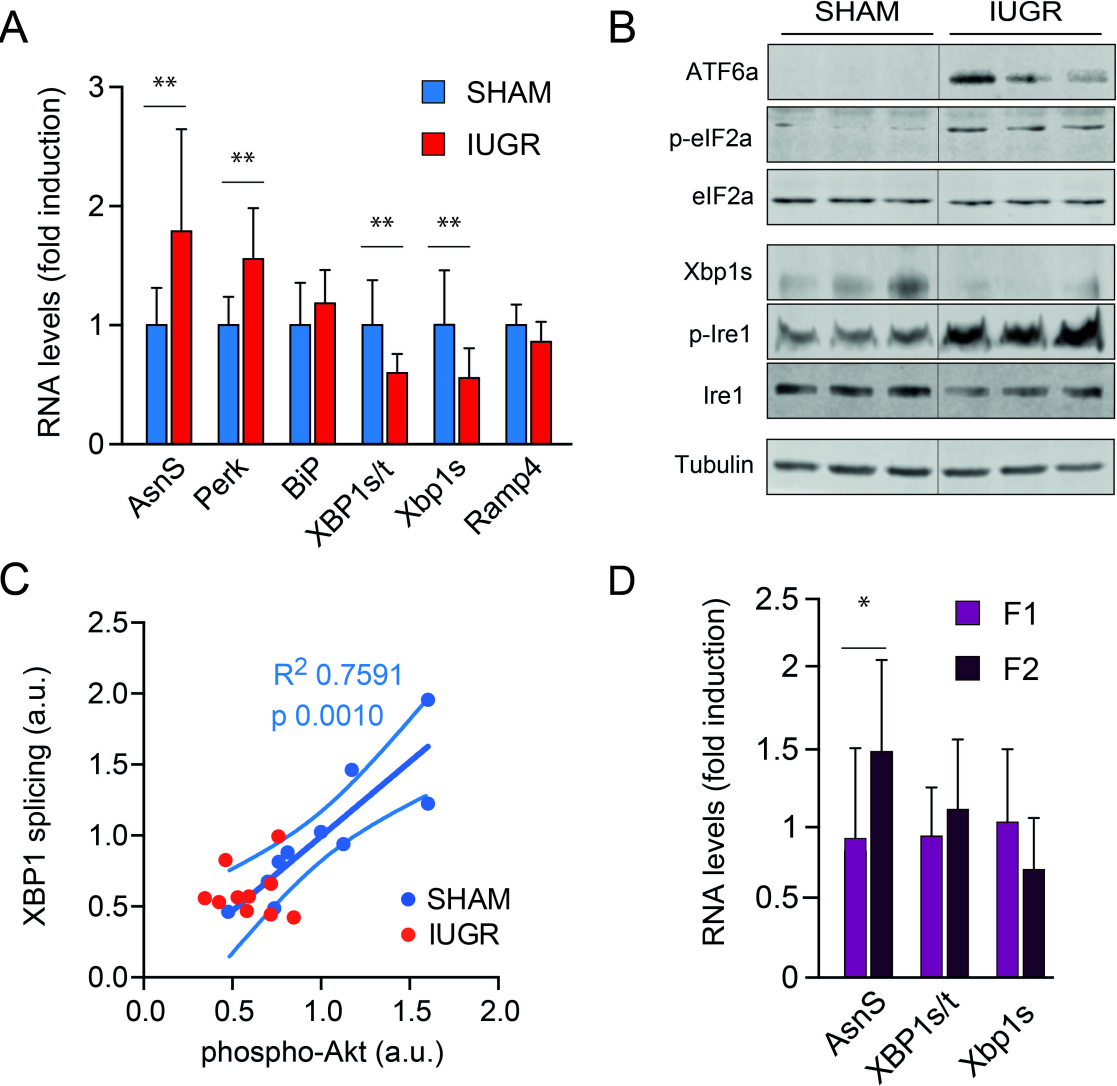
A. Expression of target genes from the three branches of UPR (IRE1 alpha, ATF6 and PERK) at PND105. Values are expressed as fold change (mean + SD, ** p<0.005). B. WIB of UPR activation (IRE1 alpha, ATF6 and PERK) at PND105. C. Relationship between mRNA Xbp1s and pAkt protein level (r = 0.86, p < 0.002) in IUGR animals. D. Relationship between liver fibrosis score (F1 and F2) and mRNA levels of UPR (IRE1 alpha and PERK), *p<0.05.

Whilst spliced XBP1 mRNA levels and Xbp1s/t ratio (0.10 ± 0.03 vs 0.17±0.07; 0.02±0.005 vs 0.04 ±0.01, respectively, p<0.01, Fig 4A) were significantly decreased in liver samples of IUGR males, PERK and ASNS were expressed at higher levels in IUGR rats (0.02 ± 0.006 vs 0.014 ± 0.003; 0.006 ± 0.002 vs 0.003±0.001, respectively, p<0.05, Fig 4A). No significant differences were observed for other UPR target genes.

Interestingly, Western blot analysis documented the activation of PERK signaling, marked by the enhanced phosphorylation of its main target, the translation initiation factor eIF2 α. Similarly, ATF6 was activated as indicated by the appearance of the 50 kDa protein corresponding to the active form of the protein (Fig 4B). Consistently with the lower levels of spliced XBP1 mRNA, XBP1s protein levels were lower in IUGR livers. IRE1α phosphorylation was increased in IUGR animals, suggesting a dysfunctional activation of signaling. A significant correlation between mRNA Xbp1s and pAkt protein t was observed in SHAM animals only (R^2^ = 0.75, p<0.01), whereas this relationship was completely lost in IUGR animals (Fig 4C). Finally, consistent with the relationship between ER stress, insulin resistance and liver fibrosis, ASNS expression, a transcriptional target of PERK, was significantly higher in animals with score 2 fibrosis when compared with score 1 animals (p<0.05) (Fig 4D).

## Discussion

In this study, we have investigated longitudinally the activation of hepatic ER stress in a rat model of intrauterine growth restriction and, at the same time, we have tested whether UPR activation is associated with metabolic dysregulation in adult animals. Our results provide the first evidence that “in utero” malnutrition stimulate UPR at birth and may lead to a dysfunctional UPR status in adulthood.

In IUGR pups, UPR target genes displayed—as a general trend–increased levels of transcription, which reached statistical significance in IRE1α branch. At the same time, both genes encoding lipogenic (Acc2, Dgat2 and Scd1) and gluconeogenic enzymes (PEPCK and G6Pase) displayed higher expression in IUGR rat pups. The transcriptional upregulation of both carbohydrate and lipid biosynthetic machineries are consistent with an acute compensatory mechanism induced by fetal exposure to malnutrition. Consistent with this, Vuguin et al. [19] have reported, in the same animal model of IUGR secondary to uteroplacental insufficiency, a modulation of the liver expression of PEPCK and glucose-6-phosphatase in IUGR animals, permanently altering liver glucose metabolism in the offspring. These changes occurred early in life before the onset of obesity and diabetes, suggesting that abnormal liver glucose metabolism may represent an early defect that contributes to the subsequent onset of fasting hyperglycemia. Our data suggest that UPR activation may be one of the mechanisms underlying the link between IUGR and dysregulation of lipid and glucose homeostasis in liver.

A growing body of evidence illustrates the close relationship between hepatic UPR and glucose and lipid metabolism. Interestingly, XBP1s promotes glucose consumption for anabolic reactions in hepatocytes, leading to hepatic glycogen storage depletion [20], and enhancing the expression of lipogenic enzymes [21,22]. On the flip side, UPR activation has been proposed to interfere with hepatic gluconeogenesis. In particular, the association of XBP1 with the transcription FOXO1 promotes its proteosomal degradation and the consequent inhibition of PEPCK and G6Pc transcription [23]. Furthermore, ATF6 disrupts the interaction of the gluconeogenic transcription factor CREB and its coactivator CRTC2, thereby reducing gluconeogenic transcription [24]. Since in the liver of IUGR animals both lipogenic and gluconeogenic genes were upregulated, we speculate that UPR signaling may serve as a counter-regulatory mechanism to mitigate the effect of the gluconeogenesis activated by in utero malnutrition thereby limiting the liver glucose output.

The early metabolism/UPR activation pattern documented in this study may reflect the ability of the organism to change structure and function in response to environmental cues, known as “developmental plasticity” [20]. Such plasticity permits a range of phenotypes to develop from a single genotype and is aimed to allow the organism to match its environment [25,26]. When environmental cues act during windows of developmental plasticity–at early phases of life–they may induce permanent changes as a result of biological “tradeoffs”. The sustained activation of UPR in adult IUGR rats stands out as one of these changes.

The histological analysis of liver from IUGR adult rats provided a clear indication of the gradual establishment of metabolic stress. Steatosis foci were scattered through the liver parenchyma. Consistent with these data, all IUGR animals showed periportal fibrosis (score 2) compared with SHAM animals. Furthermore, periportal fibrosis score was correlated with PERK activation branch. This finding is further supported by the reduced AKT phosphorylation, clearly indicating an altered insulin signaling.

We postulate that the defective insulin signaling occurs as a consequence of unresolved, chronic ER stress (PERK and ATF6 signaling activation). A crippled UPR may be insufficient to restore ER homeostasis and may promote liver steatosis [27]. Moreover, the reduction of XBP1s does not permit a complete response to stress, thereby affecting the restoration of ER homeostasis. XBP1 haploinsufficiency has been shown to drive insulin resistance in mice fed with a high fat diet ^7^. Intriguingly, IRE1α phosphorylation–a well-established indicator of IRE1 α activation–was increased in IUGR animals, suggesting a dysfunctional signaling of this UPR branch. In this scenario, the lower expression of XBP1s could contribute to unmitigated ER stress thus leading to insulin resistance. Consistent with this hypothesis was the finding of a significant correlation between Xbp1s mRNA and pAkt levels in SHAM animals only, whereas this relationship was completely lost in IUGR. Serine 724 can be phosphorylated by IRE1α *trans* autophosphorylation, but it can also be the substrate for protein kinase A (PKA). In response to fasting, PKA phosphorylates IRE1α and modulates the IRE1 capacity to stimulate hepatic gluconeogenesis [28].

Our data indicate a direct correlation between AKT phosphorylation and spliced XBP1 mRNA levels or XBP1s/XBP1 total ratio. Similarly, in vitro studies provided robust evidence for an inverse correlation between phosphorylation of eIF2α and reduced phosphorylation of Akt (Ser 473). Diet- and drug-induced stress activates–possibly through IRE1a –JNK, which in turn alters the phosphorylation status of insulin receptor substrate, and downstream insulin signaling [29–31]. In our IUGR model, a post-transcriptional downregulation of IR-beta protein levels was observed. This finding can be explained by the capacity of XBP1s to stimulate either the turnover of IR-beta or its proper folding and secretion. Alternatively, the recent identification of direct contact sites between endosomes and ER could provide an exciting mode for modulation of receptor tyrosine kinase signaling (RTKs) in the endosomal membranes [32]. Further work is needed to assess this regulation and its possible link to UPR activity.

## Conclusion

Our results show that fetal exposure to uteroplacental insufficiency is associated with activation of hepatic UPR. In parallel with IRE1α, ATF6 and PERK activation, adult male IUGR animals show an impairment of glucose tolerance and the development of hepatic steatosis with progression to fibrosis. These findings suggest that hepatic ER stress/UPR signaling may play a key role in the metabolic risk associated with intrauterine growth restriction.

This study has some limitations: a) the lack of comparison between IUGR and SHAM animals and unoperated control rats at the different time points; b) gender-related metabolic differences were not tested at 5PND; c) a longer follow-up could allow to verify the risk of overt metabolic alterations such as type 2 diabetes in adulthood; d) the evaluation of UPR in other key organs such as pancreas and adipose tissue could provide further insights into the mechanisms involved in intrauterine programming and long-term susceptibility to chronic diseases.

Further studies are needed to understand the molecular mechanisms linking the reduced nutrient supply with the activation of UPR pathways.

## Supporting information

S1 Fig Expression of main lipogenesis and gluconeogenesis genes in liver at PND105. (TIF)

S2 Fig Uncropped WIB of Fig 4. (TIFF)

S3 Fig Intrahepatic TG in 105 PND rats. No significant difference in liver TG between SHAM and IUGR rats at 105 PND was observed.
(TIFF)

S1 Table Oligonucleotide sequences of primers used in real time PCR. (DOC)
