# Supplementary figures and images for: The exposure to uteroplacental insufficiency is associated with activation of unfolded protein response in postnatal life
Source: PLoS One. 2018 Jun 13;13(6):e0198490. doi: 10.1371/journal.pone.0198490 (PMC5999290; doi:10.1371/journal.pone.0198490)

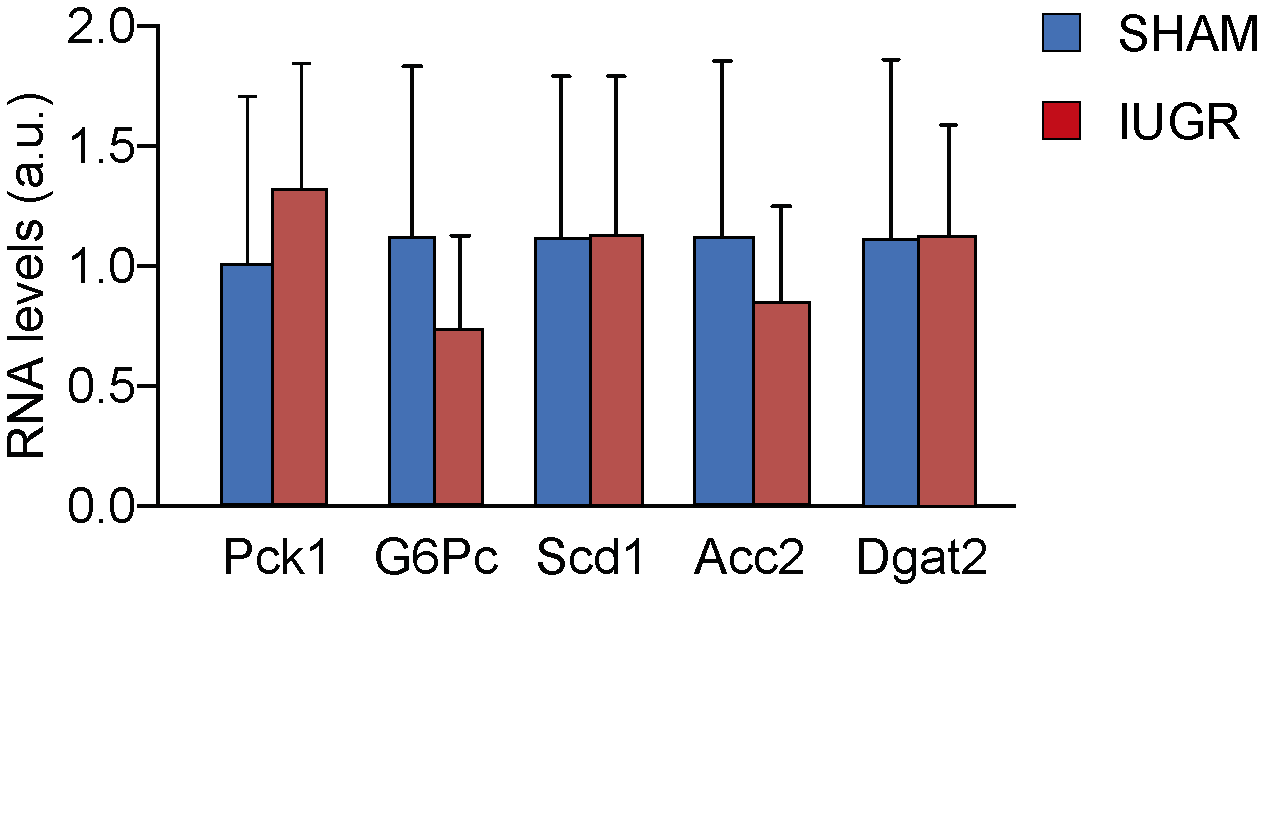

Supplement: S1 Fig — (TIF) [file pone.0198490.s001.tif]

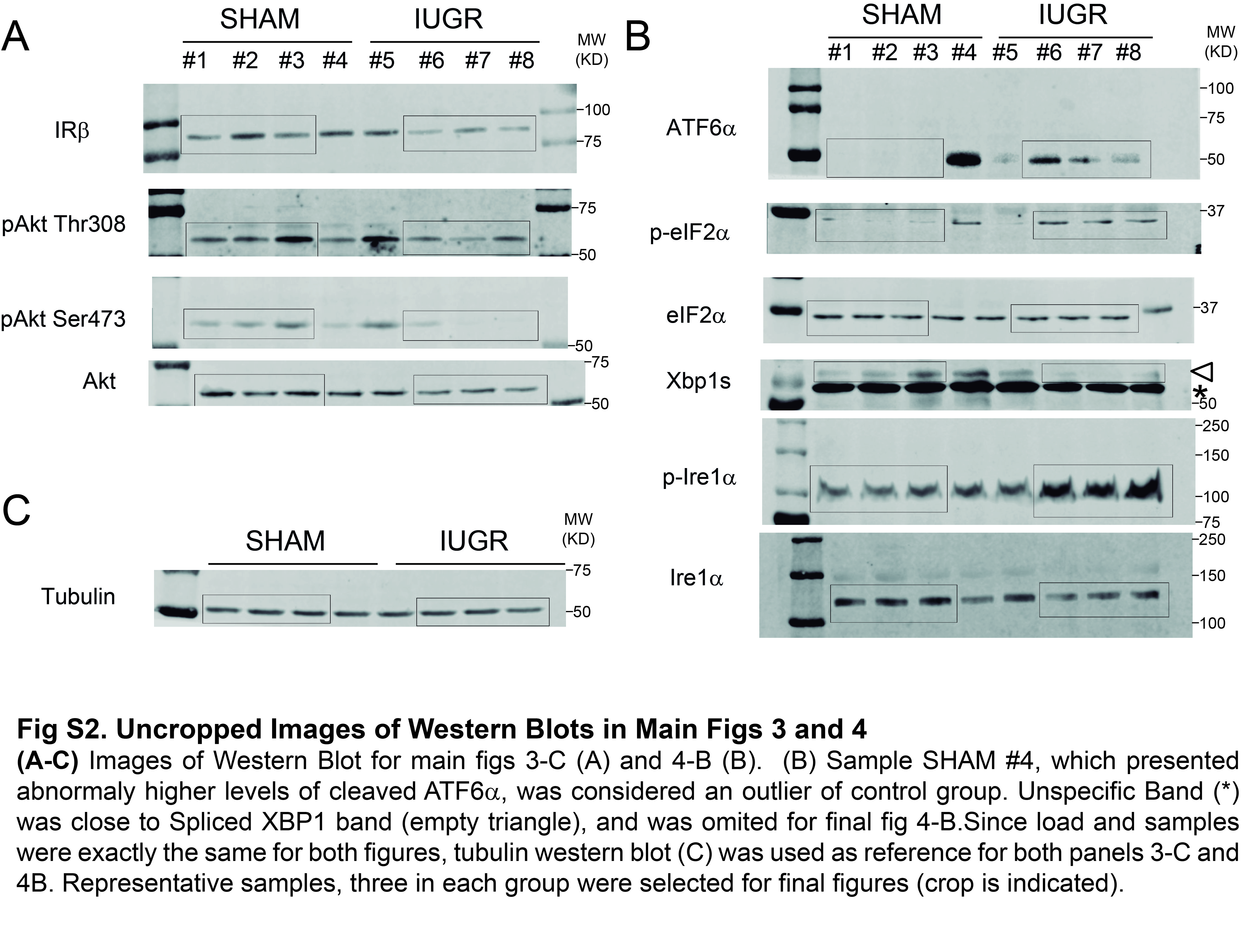

Supplement: S2 Fig — (TIFF) [file pone.0198490.s002.tiff]

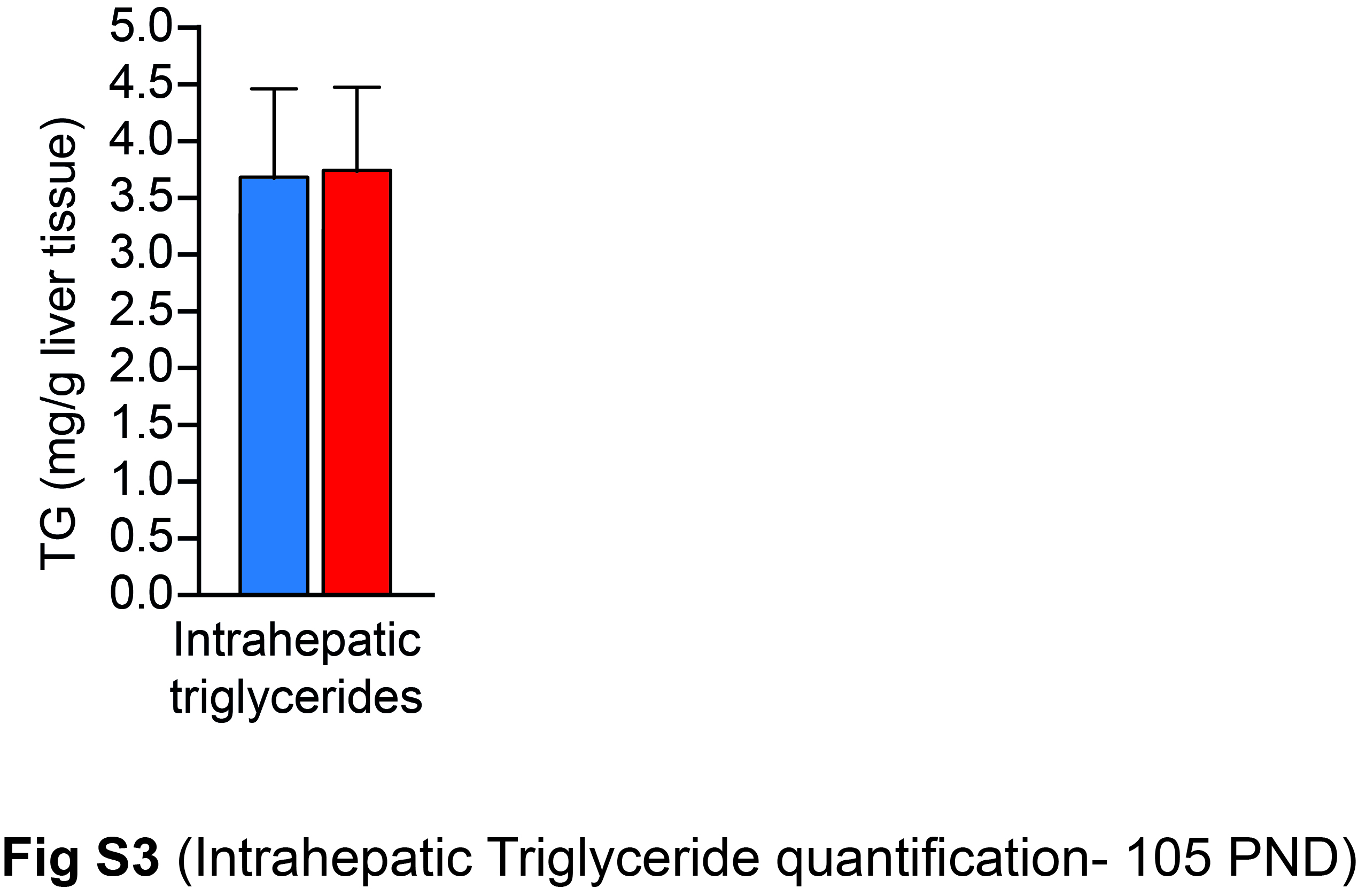

Supplement: S3 Fig — No significant difference in liver TG between SHAM and IUGR rats at 105 PND was observed. (TIFF) [file pone.0198490.s003.tiff]
