# Supplementary material for: The exposure to uteroplacental insufficiency is associated with activation of unfolded protein response in postnatal life
Source: PLoS One. 2018 Jun 13;13(6):e0198490. doi: 10.1371/journal.pone.0198490 (PMC5999290; doi:10.1371/journal.pone.0198490)
Supplement: S1 Table — (DOC) [file pone.0198490.s004.doc]

**S1 Table.** Oligonucleotide sequences of primers used in Real Time PCR*.*

| **Gene** | **Forward Primer (5’-3’)** | **Reverse Primer (5’-3’)** |
| --- | --- | --- |
| Acc2 | GTGGCCAAGAAGAACCAGCTGGTG | CGCAGCTCGTAGGAGGGGAG |
| Asns | GTATATTCGGAAGAACACAGACAGCGTGG | TTGGGTCGCCAGAGAATCTCTTTGGG |
| BiP (Grp78) | GAGCTGTGCAGAAACTCCGGCG | ACCAACTGCTGAATCTTTGGAATTCGAGT |
| Dgat2 | ACCTGGGGGCTGGTGCCCTA | GAGCTTCACCAGGGCCTCCATG |
| Dnajb9 (Erdj4) | TCACAAATTAGCCATGAAGTACCACCCTGA | GAAAACTCCTGGAAGTGATGCCTTTGTCTA |
| Eif2ak3 (Perk) | GGACTCATGCACAGGGACCTCAAGCC | GGGGGATGGAGAGAGCATGTCTTGAACC |
| Fbp1 | GTCAACTGCTTCATGCTGGACCCG | CACCATGGACCCCACATACCGG |
| G6pc | GCTGGAGTCTTGTCAGGCATTGCTG | TGGAGTTGAGGGCCAGCCCC |
| Gadd34 | AGCCCCACGCCTGAGGGCG | CAGGCGGCTCCAGGCCCG |
| Irb | GAGACACTTCACTGGGTACCGCAT | CCATTAGGTTCCTTTGGCTCTTGCCAC |
| Pck1 | GATGTGGCCAGGATCGAAAGCAAGAC | ATGATCCGCATGCTGGCCACCAC |
| Ramp4 | GGATCCGTATGGCCAACGAGAAGC | GTCAGTCACTTCACATGCCCATCCTG |
| Scd1 | TGGTGATGTTCCAGAGGAGGTACTACAA | GGTGAAGTTGATGTGCCAGCGGTAC |
| sXbp1 | CGGGTCTGCTGAGTCCGCAGCAG | CCCCACTGACAGAGAAAGGGAGG |
| tXbp1 | GCAGGTGCAGGCCCAGTTGTCAC | CCCCACTGACAGAGAAAGGGAGG |
